# Biological Crust Diversity Related to Elevation and Soil Properties at Local Scale in a Montane Scrub of Ecuador

**DOI:** 10.3390/jof9030386

**Published:** 2023-03-22

**Authors:** Leslye Ruiz, Vinicio Carrión-Paladines, Marlon Vega, Fausto López, Ángel Benítez

**Affiliations:** 1 Carrera de Biología, Universidad Técnica Particular de Loja, San Cayetano Alto s/n, Loja 1101608, Ecuador; 2 Biodiversidad de Ecosistemas Tropicales-BIETROP, Herbario HUTPL, Departamento de Ciencias Biológicas y Agropecuarias, Universidad Técnica Particular de Loja, San Cayetano Alto s/n, Loja 1101608, Ecuador

**Keywords:** diversity, lichens, bryophytes, biological soil crust, richness, elevation

## Abstract

The montane shrublands of southern Ecuador represent one of the least studied ecosystems, which in the last decade have been seriously threatened by increasing wildfires, deforestation, overgrazing, and conversion to forest plantations. Our main objective was to determine, at the local scale, the diversity of species composing the biological soil crust (BSC) at three elevations (2100, 2300, and 2500 m.a.s.l.) and their possible relationships with soil physical and chemical properties in montane shrublands. For this purpose, three monitoring plots of 100 m^2^ were established at each elevation, and within each plot, 20 subplots were established (180 subplots sampled in total). In addition, composite soil samples were collected at a depth of 0 to 10 cm, and some physical and biochemical parameters (e.g., bulk density, texture, pH, organic matter, soil organic carbon, total nitrogen, available phosphorus, and potassium) of the soil were analyzed. The results show 35 species (23 lichens, 10 bryophytes and 2 cyanobacteria) at three elevations with a bell-shaped or hump-shaped distribution pattern. This allowed us to point out that the species richness was higher at the intermediate elevations and that the composition showed significant differences in the three elevations related to soil factors. Elevation and soil drivers may help to better chose the more suitable biological soil crust (lichen-dominated and bryophyte-dominated BSC) for the management and conservation of the montane scrub of Ecuador, which is strongly threatened by human activities.

## 1. Introduction

The Ecuadorian montane scrublands (EMS), located along the inter-Andean valley, are part of the diversity hotspot known as the Tropical Andes and Tumbes–Chocó–Magdalena region [1]. The EMSs are very diverse ecosystems in flora and fauna, with endemic species that are frequently threatened by deforestation [2,3,4,5], overgrazing (cattle and goats), and conversion to eucalyptus (*Eucalyptus globulus* Labill.) and pine (*Pinus radiata* D. Don) plantations [4,5,6]. In addition, they face constant threats due to anthropogenic wildfires, which produce high and low severity fires, and the subsequent loss of soil quality due to erosion processes [7]. Despite being a highly anthropized ecosystem, the EMSs play an important role in the management of the micro-watersheds that supply drinking water to populations within Ecuador [8].

In the last decade, some studies have shown that these ecosystems allow for the establishment of organisms that make up the biological soil crust (BSC); however, the diversity of the BSC has been affected by anthropogenic activities around the world [9,10,11]. The importance of the BSC lies in the fact that it consists of a group of mosses, liverworts, lichens, cyanobacteria, fungi, and algae that inhabit the soil, and that have a close relationship with each other in order to maintain the most superficial layer of the soil [12]. 

The BSC can contribute both in terms of diversity and ecosystem processes [13]. For instance, the BSC is key for the supply of carbon (C), nitrogen (N), and other nutrients, which increase soil fertility [14,15,16,17], and supplies vascular plants and soil microbiota with essential nutrients and water [12]. Thus, these abiotic factors directly influence the diversity, abundance, and richness of the organisms that make up the BSC, which in turn allows for the presence and abundance of microorganisms and plants that benefit from their nutrients [18].

Although different studies have tried to find a relationship between the richness and composition of the species that make up the BSC with elevation, a common pattern remains unconfirmed [17,18,19]. Thus, different responses have been found, for example, increases in the richness with altitude [20] and decreases in the richness with altitude [21]. Patterns in which the species richness is bell-shaped or hump-shaped have also been observed, with the maximum richness at intermediate elevations [22,23]. However, other important factors affect the species richness and diversity in the BSC, such as the type, severity, and extent of disturbance, the vascular plant community structure, the substrate conditions [24,25], and the soil physical and chemical properties [26]. Concerning the soil properties, it is known that variables such as bulk density, porosity, texture, pH, organic matter, and carbon content can influence the species richness and BSC composition; thus, the BSC itself can modify the nutrient availability and biogeochemical cycles [27].

Globally and regionally, there have been important contributions to the knowledge of BSC composition and structure, with some research focusing on the elevation [10,28,29] and edaphic and environmental variables [27,30]. Most of this research has been conducted in arid and semi-arid regions in subtropical areas of the United States, Spain, and Australia [31,32,33], as well as in temperate regions [34], while in tropical regions, such as Mexico and southern Africa, little research has been conducted on the BSC [35,36]. On the other hand, studies in South America have been conducted in Chile, Brazil, Argentina, and Venezuela [11,16,37,38]. In Ecuador, the diversity of the BSC in relation to the elevation was analyzed in the dry scrubland in the south of the country [17,20]. In this context, it is necessary to generate new scientific data to understand the ecological role of the BSC in the EMSs and their relationships with the elevation and the physical and chemical properties of the soil. These data would serve to better understand the potential benefits of the BSC in the anthropized EMSs of southern Ecuador. 

The objective of this research was to determine the diversity of the species that make up the BCS at three elevations of the EMS of southern Ecuador (2100, 2300, and 2500 m.a.s.l.) and its possible relationships with the physical and chemical properties of the soil. The results of this research allowed for the acquisition of valuable information that will help in the conservation of this type of highly anthropized ecosystem, which is currently facing biodiversity loss.

## 2. Materials and Methods

### 2.1. Study Area

**Figure 1:**
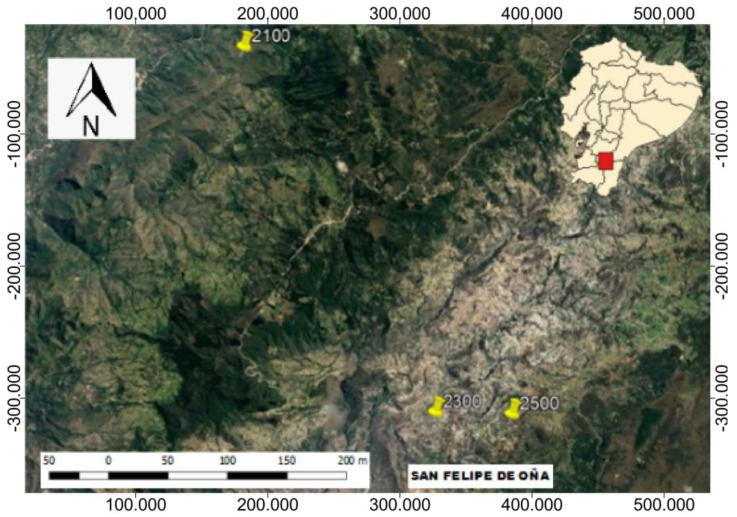
Study area (Cantón Oña-Azuay province) with the three elevations in the montane shrublands of southern Ecuador.

The study was carried out in an EMS located in Oña canton, Azuay province (Figure 1). The climate of Oña canton is warm temperate with an annual minimum temperature of 12 °C and an annual maximum temperature of 20 °C. There is year-round rainfall of between 1000 and 3000 mm/year [39,40]. The southern EMS is characterized by a vegetation cover dominated by the species of the genera *Croton, Cortaderia, Pennisetum, Baccharis, Acacia,* and *Agave* [5].

**Figure 2:**
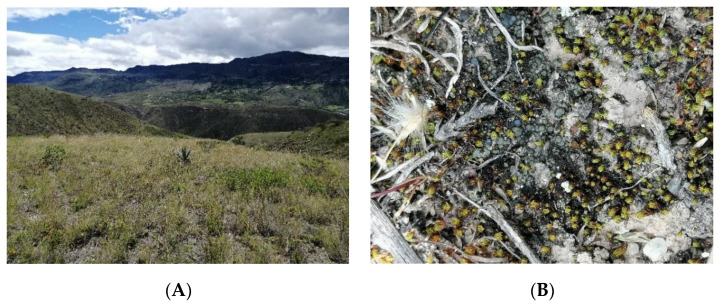
(**A**). The montane shrublands of southern Ecuador; (**B**). Mixed biological soil crust (lichen-dominated and bryophyte-dominated BSC).

### 2.2. Design and Data Collection

Three elevations were selected in the EMS, with a nested design, each separated by ± 200 m (Elevation 1: 2100 m.a.s.l.; Elevation 2: 2300 m.a.s.l. and Elevation 3: 2500 m.a.s.l.) (Figure 2A). At each elevation, it was not possible to carry out replicates because the studied shrubland is an ecosystem that has little surface area due to the effects of the reconversion to which it has been subjected in recent decades [7], which also limited the logistics for sampling. Three 10 m × 10 m plots (9 plots in total), evenly spaced at a distance of 10 m each, were established at each elevation. Within each plot, 20 quadrants of 30 cm × 30 cm were established and divided into grids of 5 cm × 5 cm, for a total of 60 quadrants for each elevation. The subplots were randomly distributed to ensure the presence and cover of the BSC, with a minimum separation of 1.5 m to reduce the risk of obtaining erroneous or dependent information [20]. We used the estimated cover as a surrogate of species abundance because cover is a good estimator of biomass for BSC [17]. The specimens of lichens and bryophytes were identified using numerous published keys. For the lichens, standard microscopy techniques and spot tests based on thallus fluorescence under ultraviolet light (UV), reactions with K (10% water solution of potassium hydroxide), C (commercial bleach), and Lugol’s solution (I) were checked in the species. For the nomenclature of the lichen species, we followed MycoBank (www.mycobank.org/ (accessed on 1 January 2022) and LIAS 1995–2016 (A Global Information System for Lichenized and Non-Lichenized Ascomycetes: www.lias.net (accessed on 1 January 2022), and for the bryophytes, the Liverworts and Hornworts of Colombia and Ecuador and Catalogue of the Plants and Lichens of Colombia. Specimens were deposited in the Herbarium of Universidad Técnica Particular de Loja (HUTPL, Loja, Ecuador).

### 2.3. Soil Sampling

Three random samples were taken from each sample plot for the determination of the bulk density (gr cm^−1^) and another three random samples for the texture and chemical analysis (e.g., pH). The sampling depth was 0–10 cm for both the bulk density and texture and chemical analysis samples. Each sample was collected using standardized metal cores (6 cm diameter, 10 cm height, 283 cm^3^ volume), as recommended by Munkholm et al. [41] and Guzmán et al. [26]. The bulk density samples were duly separated and labeled (3 per plot, 9 per elevation, 27 in total), while the other 3 samples from each plot, for the textural and chemical analysis, were mixed to obtain a composite sample (1 composite sample per plot, 3 per elevation, 9 samples in total).

The bulk density was determined by the cylinder method, for which the individual samples were dried in an oven for 48 h at 105 °C [42]. The soil porosity was determined using the soil particle density assumption corresponding to 2.65 g cm^3^ [43]. The samples for the determination of the texture, pH, and macro and micronutrient content were dried at room temperature for 72 h. Subsequently, all visible roots were removed and the samples were sieved through a 2 mm mesh. The soil texture was determined by the Bouyoucos method [44]; the pH was measured with a pH meter using the standard method [44]. The soil organic carbon (SOC) and soil organic matter (SOM) were determined using the Walkley and Black method [45], for which the sample was placed in an oven at 125 °C for 45 min, after oxidation in a K_2_Cr_2_O_7_/H_2_SO_4_ solution. The total nitrogen (TN%) was determined by the Kjeldahl method, the phosphorus content (mg/kg) by the modified Olsen method [46], and the potassium (cmol/kg), Ca (cmol/kg), Mg (cmol/kg), Fe (mg/kg), Mn (mg/kg), Cu (mg/kg), and Zn (mg/kg) contents by atomic absorption spectrophotometry [47].

### 2.4. Data Analysis

The diversity of the biological soil crust was analyzed by means of species richness and cover; we also estimated the species richness of the BSC by elevation with accumulation curves and the Chao 2 richness estimator [48].

The soil physicochemical analysis data from the different contrasting elevations were subjected to a one-way analysis of variance (ANOVA, F-test, *p* < 0.05). A normality test (Shapiro-Wilk) was performed on all of the mean values before applying the parametric tests.

We used generalized linear models (GLM) to analyze the effects of elevation and soil factors on the richness and cover of the BSC; in the GLM, we used Poisson distribution as the error distribution and log link function [49]. The species composition related to elevation and edaphic properties were evaluated through a non-metric multidimensional scaling analysis (NMDS) using Euclidean distance and 999 Monte Carlo permutations. To analyze the effect of elevation and soil variables (e.g., bulk density, porosity, texture, and chemical analysis), a permutation-based multivariate analysis (PERMANOVA) was performed. All of the analyses were performed in the statistical program R 3.2.2. and the statistical package “vegan” [50].

## 3. Results

### 3.1. Alpha Diversity

A total of 35 species were recorded, including 8 mosses, 2 liverworts, 2 cyanobacteria, and 23 lichens (Table 1). The biological soil crust are heavily dominated by lichens and mosses (lichen-dominated, and bryophyte-dominated crusts, Figure 2B).

**Figure 3:**
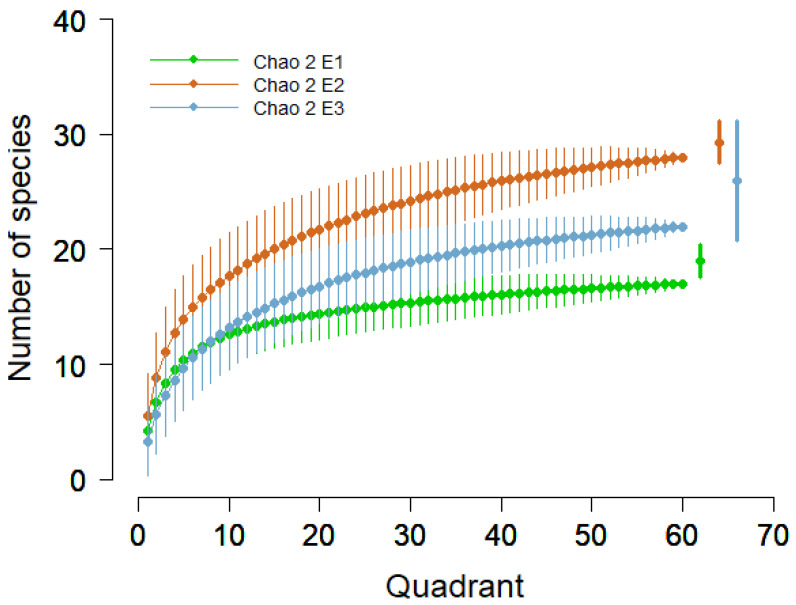
Accumulation curves with 95% confidence intervals and Chao 2 estimator (points on the right of the figure) for three elevations.

Within the first elevation (E1 2100 m), 19 species were found (4 mosses, 2 cyanobacteria, and 13 lichens), while in the second elevation (E2 2300 m) 28 species were found (6 mosses, 1 liverwort, 1 cyanobacterium, and 20 lichens) and in the third elevation (E3 2500 m), 24 species (6 mosses, 2 liverworts, and 16 lichens) were found. The accumulation curves and Chao 2 richness estimator showed high values of estimated species for the E2, follow E3 and E1, respectively (Figure 3).

**Figure 4:**
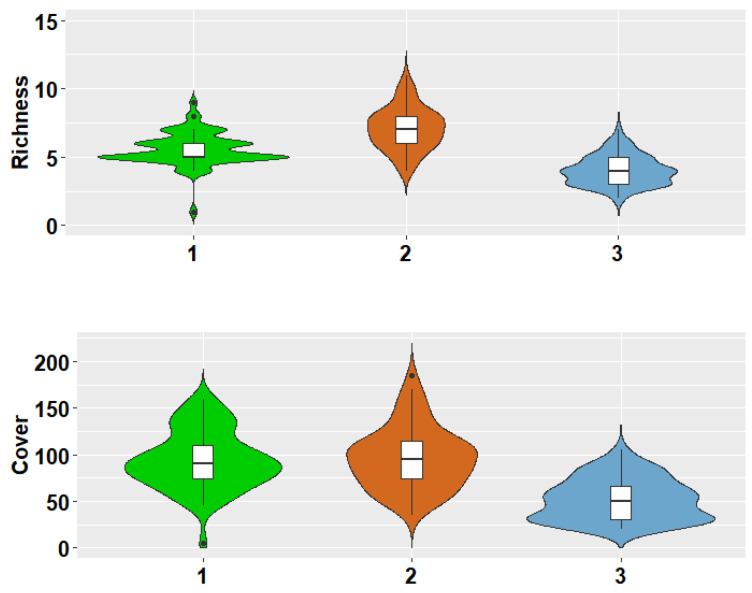
Violin diagram of species richness and cover in three elevations. In each violin diagram, a box plot showing the distribution of the data can be observed, followed by a thin line representing the confidence interval, while the width of the graph indicates the frequency of the species in each elevation.

The violin plot showed that the highest species richness and cover of the biological soil crust were found in the highest elevations, with a bell-shaped or hump-shaped distribution pattern (Figure 4).

### 3.2. Characteristics of the Physical-Chemical Properties of the Soil in Three Elevations

The bulk density (Bd), soil porosity, sand, silt and clay showed no significant statistical differences at three elevations (Table 2). The chemical properties of the soils of each contrast elevation (depth 0–10 cm) showed statistical differences in the pH, K, Mg, Ca, and Fe, while for the SOM (%), N, P, SOC (%), C/N ratio, Mn, Cu, and Zn, no statistically significant differences were observed (Table 2).

The generalized linear model (GLM) indicated that richness and cover are influenced by the elevation and by edaphic properties such as the pH, bulk density, K, Fe, and SOM content, where E1 and E2 had a positive influence and E3 had a negative effect, thus forming a bell-shaped pattern (Table 3).

### 3.3. Beta-Diversity

**Figure 5:**
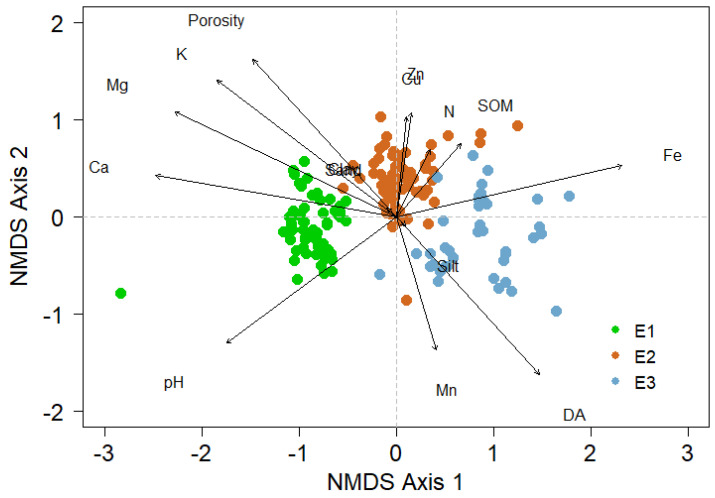
NMDS ordination bi-plots of quadrants scores, the environmental and soil vectors fitted in the ordination space. The ordination is based on species cover data of biological soil crust in the three elevations (E1–E3).

NMDS analysis indicated a clear clustering of BSC species related to conditions at different elevations (Figure 5).

The elevation influenced the composition of the biological soil crust communities (Table 4), explaining 35% of the variability in the species composition. Similarly, an effect of the edaphic properties was noted, where the bulk density, K, Fe, pH, and SOM are the main drivers of BSC diversity.

## 4. Discussion

### 4.1. Alpha and Beta Diversity of BSC at Three Elevations

The results of this study constitute the first report on the diversity of the BSC in an EMS, and the relationship between the elevation and soil factors at the local scale. The richness and cover of the BSC showed a bell-shaped or hump-shaped trend. These results are consistent with those reported by Grytnes et al. [23] and Baniya et al. [51], who documented a bell, curve, or hump shape of the BSC species richness, which were influenced by the elevation. In contrast, a study in Ecuador in dry scrublands indicated that the richness increased with the elevation [20]; however, where in that study, a smaller gradient (of 100 m between sampling elevations) was considered, in our study, a larger distance between elevations (of 200 m) was considered. Similarly, studies have been conducted focusing individually on the different groups that make up the BSC concerning elevation; for example, in lichens [22,52] and bryophytes [29,53], a bell-shaped or hump-shaped pattern was also obtained.

On the other hand, the species composition is different at the three elevations, similar to the BSC results in dry shrubland [17]. In agreement with our results, E3 is dominated mainly by bryophytes (e.g., *Campylopus richardii, Fossombronia peruviana*, *Leptodontium viticulosoides*) as these tend to grow in and colonize higher elevation areas due to the ability to retain water and contribute to increased organic matter, with the exception of *Bryum argenteum*, which tends to grow at low elevations, as evidenced in our study [17,54]. Regarding lichens, we reported a higher diversity and cover at the 2300 m and 2500 m elevations (e.g., *Cladonia rappi*, *Coccocarpia palmicola*, *Diplochistes diacapsis*, *Lepraria* aff. *diffusa*, *Psora* aff. *pruinosa*, *Cladia aggregata*), which coincides with the other studies [55]. However, according to Concostrina-Zubiri et al. [56], there are exceptions to this, such as the species *Xanthoparmelia subplittii* y *Xanthoparmelia mougeotii*, whose growth develops at low elevations or in areas intended for grazing; this result is similar to that obtained in this study. On the other hand, the presence of cyanobacteria in our study occurs at low altitudes, where, similarly, Blay et al. [57] point out that cyanobacteria decrease in high elevations, where there is a dominance of other organisms that make up the BSC (mosses and lichens) and vascular plants [58]. Similarly, cyanobacteria tend to grow in sandy soils and are resistant to extreme conditions of drought, temperature, and humidity [12].

### 4.2. Relationships between Diversity of BSC and Soil Properties

Other factors that affect the species richness and composition of the BSC are related to the physical-chemical properties of the soil [59]. In our case, bryophyte species [60], such as *Bryum argenteum* and *Syntrichia* sp., and lichen species such as *Lepraria* aff. *diffusa*, *Psora icterica*, *Toninia* aff. *Submexicana*, and *Xanthoparmelia mougeotii* were not affected by the soil texture. According to Kalníková et al. [61] and Hansen and Goertzen [62], these species, and especially those of the genera *Bryum* and *Psora*, grow adequately in soils with a sand concentration (sandy soils).

On the other hand, the bulk density was lower in E1, where *Bacidia* sp., *Nostoc commune*, and *Peltula obscurans* var *obscurans* were the exclusive species for this elevation, which may be benefiting from the low value of bulk density. This is because, when there is more pore space (E1: 67.2% higher porosity), soils generally do not have compaction problems, as shown by recent research [63]. In E2 and E3, clay and silt are higher than in E1 (and sand is lower), which is in agreement with previous studies that have shown that a high percentage of fine material in the soil is important for the development and diversity of the BSC [35,64]. In addition, the diversity of the BSC protects the soil by preventing raindrops from directly impacting the surface and thereby dislodging soil particles [63] and anchoring structures, such as lichen rhizines and moss rhizoids, which are physically attached to the soil particles [65].

The species composition of the BSC showed a significant correlation with the pH, Ca, and Mg in E1. This result was expected as several species belonging to the genera *Bacidia*, *Nostoc*, and *Peltula* have been commonly associated with neutral pHs [17]. Similarly, for E2, the K, Cu, Zn, SOM, and porosity were correlated, while for E3, the SOM, Fe, and bulk density corresponded. The SOM contents in the three elevations presented low concentrations (<2%); [66], while nitrogen values, in E1 and E2, are considered medium, and in E3, it is considered medium to high (<0.12%); [67]. This same pattern is observed for the carbon contents, where in all three elevations, there is a low concentration, corresponding to low fertility (<1.2% SOC [66,68]). However, despite the low soil quality, the presence of SOC represents an important contribution of carbon (C) and nitrogen (N) to the soil, as demonstrated by some research [31,69]. This is because the presence of the BSC increases the soil stability and protects it against the erosive action of rain and wind, and directly affects the establishment, nutritional content, and water availability for vascular plants [12,31,69].

Finally, Bowker et al. [70] demonstrated that the concentration of macro- or micro-nutrients influences the richness and composition of the BSC; such is the case of lichens, which are associated with soils that present high levels of N, C, and P. Other studies mention that BSC dominated by lichens and bryophytes prefer soils with good concentrations of Mn, Zn, K, Mg, Fe, and Ca [69]. However, other researchers, such as Ochoa-Hueso et al. [71], report that the concentration of Mn and Zn can affect the composition of lichens. In this context, we recommend further research specifically focusing on the effect of micronutrients or the effect of the BSC on micronutrients to elucidate this issue.

## 5. Conclusions

The diversity and composition of the biological soil crust is influenced by the elevation and the physical and chemical properties of the soil at the local scale, with a bell-shaped or hump-shaped distribution pattern. We found that the texture does not limit the diversity and cover of BSC species; in contrast, the bulk density, pH, silt, and clay contents at higher elevations (E2 and E3) affect the richness and composition of the BSC. Thus, the diversity of the BSC serves as a regulator of the SOM content, providing nutrients to the soil and to the microorganisms that are part of this system. The loss of BSC by human activities (e.g., fires, deforestation) can lead to a loss of soil stability and increased soil infertility by obstructing the fixation of carbon, nitrogen, and other nutrients from the BSC to the soil. The diversity of the biological soil crust (lichen-dominated and bryophyte-dominated BSC) can be used as a bioindicator of environmental factors (elevation and soil factors) in the montane scrub of Ecuador.

## Figures and Tables

**Table 1:** Lichen, bryophyte and cyanobacteria species and number of subplots on which each species appears in the three elevations in an EMS in southern Ecuador.

Species	E1 (2100 m.a.s.l.)	E2 (2300 m.a.s.l.)	E3 (2500 m.a.s.l.)
**Mosses**			
*Bryum argenteum* Hedw.	30	4	7
*Bryum* sp.	0	3	0
*Campylopus richardii* Brid.	0	0	26
*Campylopus* sp.	0	3	7
*Leptodontium viticulosoides* (P.Beauv.) Wijk & Margad.	0	4	35
*Syntrichia andicola* (Mont.) Ochyra	1	0	0
*Syntrichia* sp.	55	53	25
*Weissia* sp.	21	55	20
**Liverworts**			
*Fossombronia peruviana* (Gottsche & Hampe)	0	1	4
*Gymnomitrion* aff. *bolivianum* (Steph.) Váňa	0	0	1
**Cyanobacteria**			
*Nostoc commune* Vaucher ex Bornet & Flahault	42	0	0
*Scytonema* sp.	58	36	0
**Lichens**			
*Bacidia* sp.	1	0	0
*Buellia* sp.	2	1	0
*Caloplaca* sp.	0	3	0
*Cladia aggregata* (Sw.) Nyl.	0	9	24
*Cladonia* sp.	0	6	10
*Cladonia* sp1	0	25	17
*Cladonia rappii* A.Evans	0	17	7
*Coccocarpia palmicola* (Spreng.) Arv. & D.J.Galloway	0	26	8
*Cora glabrata* (Spreng.) Fr.	0	8	2
*Diploschistes diacapsis* (Ach.) Lumbsch	0	47	4
*Flakea* sp.	0	46	58
*Lepraria* aff. *diffusa* (J.R. Laundon) Kukwa	2	45	11
*Lepraria* sp.	14	9	1
*Leptogium* sp.	15	7	2
*Peltula obscurans* var *obscurans* Wetm.	1	0	0
*Placidium squamulosum* (Ach.) Breuss	28	15	0
*Psora* aff. *pruinosa* Timdal	0	29	5
*Psora icterica* (Mont.) Müll.Arg.	9	3	4
*Toninia* sp.	3	11	0
*Toninia* aff. *submexicana* B.de Lesd.	17	1	1
*Toninia wetmorei* Timdal	16	0	4
*Xanthoparmelia subplittii* Hale	14	2	0
*Xanthoparmelia mougeotii* (Schaer.) Hale	52	19	3

**Table 2:** Results of one-way ANOVA of main physical-chemical properties of the soil at the different elevations studied.

Properties	E1 (m.a.s.l. 2100)	E2 (m.a.s.l. 2300)	E3 (m.a.s.l. 2500)	*p*-Value
Bulk density (g cm^−3^)	0.87 ± 0.02	0.91 ± 0.03	1.05 ± 0.13	0.061
Porosity (%)	67.2 ± 0.75	65.7 ± 1.00	60.4 ± 4.86	0.062
Sand (%)	59.9 ± 5.77	62.1 ± 1.91	62.6 ± 0.42	0.632
Silt (%)	14.0 ± 3.46	12.7 ± 1.15	12.4 ± 0.26	0.633
Clay (%)	26.1 ± 2.31	25.2 ± 0.75	25.0 ± 0.15	0.632
Textural class	Sandy clay loam	Sandy loam	Sandy clay loam	--
pH	7.3 ± 0.2	5.7 ± 0.2	6.1 ± 0.2	0.000
SOM (%)	1.6 ± 0.1	1.8 ± 0.1	1.9 ± 0.6	0.592
N (%)	0.09 ± 0.01	0.09 ± 0.01	0.10 ± 0.03	0.798
P (mg/kg)	3.5 ± 0.00	3.5 ± 0.00	3.5 ± 0.00	0.100
K (mg/kg)	0.49 ± 0.08	0.42 ± 0.08	0.24 ± 0.09	0.025
SOC (%)	0.94 ± 0.05	1.04 ± 0.06	1.11 ± 0.34	0.602
C/N ratio	10.8 ± 0.4	11.1 ± 0.00	11.5 ± 0.4	0.091
Mn (mg/kg)	4.4 ± 0.37	2.9 ± 1.27	5.5 ± 1.13	0.053
Mg (cmol/kg)	2.7 ± 0.12	1.8 ± 0.39	0.9 ± 0.29	0.000
Ca (cmol/kg)	10.6 ± 0.94	6.3 ± 0.42	4.2 ± 1.66	0.001
Fe (mg/kg)	24.8 ± 5.30	81.9 ± 11.25	87.3 ± 8.00	0.000
Cu (mg/kg)	1.8 ± 0.07	3.4 ± 3.57	1.7 ± 0.79	0.571
Zn (mg/kg)	1.6 ± 0.00	2.0 ± 0.66	1.6 ± 0.00	0.421

**Table 3:** Generalized linear models (GLM) to explore effects in richness and cover of biological soil crust related to elevation and soil factors.

Richness				
Estimate	Coefficient	Std. Error	T	*p*-Value
E1	−21.1277	5.6888	−3.7140	0.0003
E2	3.4233	0.8211	4.1690	0.0000
E3	2.4856	0.7452	3.3350	0.0010
pH	3.6763	0.9122	4.0300	0.0001
DA	−5.0010	1.3484	−3.7090	0.0003
K	2.8071	0.7409	3.7890	0.0002
Fe	0.0624	0.0157	3.9650	0.0001
SOM	−1.6713	0.4026	−4.1510	0.0001
**Cover**	**Coefficient**	**Std. Error**	**T**	***p*-Value**
E1	−23.9494	9.1420	−2.6200	0.0096
E2	3.4135	1.3150	2.5960	0.0103
E3	2.3121	1.2073	1.9150	0.0571
pH	4.6722	1.4855	3.1450	0.0020
DA	−7.2094	2.3819	−3.0270	0.0029
K	3.2673	1.2429	2.6290	0.0093
Fe	0.0907	0.0262	3.4660	0.0007
SOM	−2.0850	0.6731	−3.0980	0.0023

**Table 4:** Results of PERMANOVA analysis of species composition of biological soil crust by elevation and soil factors of the EMS of southern Ecuador. df = degrees of freedom; SS = sum of squares; MS = median squares, F = F-statistics; R^2^ = coefficient of variation.

Factors	df	SS	MS	F	R^2^	*p*-Value
Elevation	2	21.677	10.8385	65.305	0.38918	0.001
Bulk density	1	1.724	1.7244	10.39	0.03096	0.001
K	1	1.236	1.2359	7.446	0.02219	0.001
Fe	1	1.233	1.2335	7.432	0.02215	0.001
pH	1	0.574	0.574	3.459	0.01031	0.006
SOM	1	0.397	0.3966	2.39	0.00712	0.025
Residuals	171	28.38	0.166		0.50953	
Total	179	55.699			1	

## Data Availability

Not applicable.
